# In-House Fabricated One-Step SPE Cartridge with HPLC-MS/MS for Fast and Accurate Determination of Multi-Class Contaminants in Eggs

**DOI:** 10.3390/foods15152716

**Published:** 2026-08-01

**Authors:** Hongyan Zhang, Qiaoying Chang, Yun Li, Tao Peng, Bo Zhao

**Affiliations:** 1 Key Laboratory of Pesticide and Veterinary Drug Monitoring for State Market Regulation, Lanzhou Institute for Food and Drug Control, Lanzhou 730050, China; 2 Chinese Academy of Quality and Inspection & Testing, Beijing 102600, China

**Keywords:** eggs, one-step SPE, veterinary drugs, pesticides, mycotoxins, HPLC-MS/MS, response surface methodology

## Abstract

Eggs are highly susceptible to simultaneous contamination by veterinary drugs, pesticides and mycotoxins across the entire laying hen production chain, yet the quantitative analysis of these multi-class contaminants with a wide polarity span in egg matrices remains challenging. In this work, an in-house fabricated one-step SPE cartridge coupled with high-performance liquid chromatography-tandem mass spectrometry was established for their simultaneous, accurate and sensitive quantification. Response surface methodology was adopted to optimize the sorbent composition of the self-made cartridge. All target analytes achieved correlation coefficients (R^2^) higher than 0.99 over their respective linear ranges. The limits of detection and limits of quantification (LOQs) spanned 0.1–3 μg/kg and 0.2–10 μg/kg, respectively. Matrix effect (ME) assessment revealed weak ME for 60% of the compounds. At three fortification levels (LOQ, 2× LOQ and 10× LOQ), recoveries of all 60 analytes fell within 70–120%, with relative standard deviations lower than 20%. The above results confirm that the proposed method can simultaneously quantify veterinary drugs, pesticides and mycotoxins; greatly shorten sample pretreatment duration; and markedly improve overall analytical efficiency.

## 1. Introduction

Eggs are one of the most widely consumed animal-derived protein foods worldwide. Rich in high-quality amino acids, lecithin and trace elements, they serve as a core nutritional component in people’s daily diets. However, multiple chemical contamination risks run through the entire laying hen breeding chain. During feeding management, veterinary drugs including quinolones, sulfonamides, tetracyclines and amphenicols are often abused to control poultry diseases and increase egg yield, easily resulting in drug accumulation and persistent residues in eggs [1]. Pesticides such as insecticides and fungicides sprayed during feed crop cultivation can transfer and bioaccumulate in eggs via the food chain [2]. Furthermore, mycotoxins from moldy feed will remain in eggs as parent toxins or in their metabolites after ingestion by laying hens, and eventually enter the human dietary chain [3]. Long-term consumption of egg products containing excessive contaminants can induce intestinal flora disturbance, proliferation of drug-resistant bacteria, and chronic liver and kidney damage in humans. Moreover, some residual pollutants possess teratogenic, carcinogenic and mutagenic properties. The co-occurrence of multiple contaminants may trigger synergistic toxicological effects, which poses a serious threat to public food safety and human health [4,5,6].

Eggs constitute a complex biological matrix that demands rigorous and efficient sample pretreatment. Primarily composed of water, egg white and egg yolk, egg matrices contain abundant endogenous interfering substances. Specifically, egg white is rich in proteins, vitamins and trace lipids, while egg yolk contains high levels of lipids, minerals, and fat-soluble vitamins (A, D, E, and K) [7,8]. During mass spectrometric detection, co-extracted lipid impurities are the main cause of unstable signal reproducibility and severe electrospray ionization (ESI) ion suppression [9]. Accordingly, reliable and accurate analytical results highly depend on elaborate pretreatment protocols with efficient cleanup procedures to eliminate matrix interferences and obtain high-purity extracts for instrumental analysis [10].

Common sample pretreatment strategies for the analysis of trace contaminants in food matrices include liquid–liquid extraction [11], solid–liquid extraction [12], QuEChERS [13,14,15,16,17], and solid-phase extraction (SPE) [18]. Nevertheless, conventional QuEChERS extracts overwhelmed the sorbents in the mini-cartridges in most cases, leading to less cleanup and worse performance in the most complex matrices [15]. Although novel adsorbents such as Enhanced Matrix Removal-Lipid (EMR-Lipid) have been developed to alleviate matrix interference, their applicability for multi-class contaminant screening remains limited. In addition, traditional cartridge-based SPE requires time-consuming sequential operations, including conditioning, sample loading, washing, and elution, which involve high organic solvent consumption and low batch-processing throughput [19]. Furthermore, most existing analytical protocols are restricted to the separate detection of veterinary drugs, pesticides and mycotoxins. Although high-performance liquid chromatography coupled with quadrupole time-of-flight mass spectrometry has been applied for multi-residue screening of veterinary drugs and pesticides [20], few methods enable the simultaneous and efficient quantification of broad-polarity analytes. It remains challenging to achieve unified extraction and cleanup for highly polar fipronil and its metabolites, moderately polar quinolones, and ultra-trace mycotoxins in a single analytical run, greatly restricting the integrated monitoring of combined egg contamination.

To address these limitations, we herein developed and validated an in-house fabricated one-step SPE cartridge coupled with HPLC-MS/MS for the simultaneous, accurate and sensitive determination of multi-class residues, including veterinary drugs, pesticides and mycotoxins in eggs. This method was applicable to 60 target analytes spanning 13 categories with vastly divergent physicochemical properties, covering a broad log P (octanol–water partition coefficient) range from −2.2 to 7.4. The target analytes screened in this study were selected according to the Rules for the Implementation of Food Safety Supervision Sampling Inspection of China (2026 Edition), combined with high-risk residual compounds frequently detected in egg matrices. Response surface methodology (RSM) was adopted to optimize the sorbent composition of the self-made cartridge. Furthermore, the established analytical approach was successfully applied to the screening of real commercial egg samples and exhibits promising prospects for routine food safety risk assessment.

## 2. Materials and Methods

### 2.1. Materials and Reagents

Agilent 1290 Infinity II LC-6460 Triple Quadrupole Liquid Chromatography-Mass Spectrometer, coupled with a chromatographic column of SB-C18 (100 mm × 2.1 mm ID, 3.5 μm particle size) (Agilent, Santa Clara, CA, USA), was used. The following were used: contaminant standards (Alta Technology Co., Ltd., Tianjin, China), ammonium acetate (Fisher, Pittsburgh, PA, USA, 97%), chromatographical grade acetonitrile (ACN) (Fisher, Pittsburgh, PA, USA), chromatographical grade MeOH (methanol) (Fisher, Pittsburgh, PA, USA), chromatographical grade formic acid (FA) (Fisher, Pittsburgh, PA, USA, 99%), analytical grade glacial acetic acid (AA) (Longxi Science, Longxi, China, 99.5%), anhydrous sodium acetate and anhydrous magnesium sulfate (Agilent, Santa Clara, CA, USA), primary secondary amine (PSA) (Agela, Tianjin, China), graphitized carbon black (GCB) (Agela, Tianjin, China), C18 (Agela, Tianjin, China), 10 mL polypropylene cartridges (80 mm × 16 mm ID) (ANPEL, Shanghai, China), polyethylene fitted SPE (16 mm ID) (ANPEL, Shanghai, China). The chemical information of 60 analytes was displayed in Table S1.

### 2.2. Instrumentation and Instrumental Conditions

Agilent 1290 Infinity II LC-6460 Triple Quadrupole Liquid Chromatography-Mass Spectrometer, coupled with a chromatographic column of SB-C18 (100 mm × 2.1 mm ID, 3.5 μm particle size). The column temperature was set to 40 °C. In the positive-ion mode, the mobile phase was formed with 0.1% FA aqueous solution (A) (containing 5 mmol/L ammonium acetate) (*v*/*v*), and ACN (B). The flow rate was 0.3 mL/min. The gradient elution program was programmed as follows: 0–3 min, 5–10% B; 3–8 min, 10–20% B; 8–11 min, 20–30% B; 11–14 min, 30–50% B; 14–16 min, 50–70% B; 16–17 min, 70–90% B; 17–17.01 min, 90–5% B; 17.01–20 min, 5% B. In the negative-ion mode, the mobile phase was formed with aqueous solution (A) (containing 5 mmol/L ammonium acetate) (*v*/*v*), and ACN (B). The flow rate was 0.3 mL/min. The gradient elution program was programmed as follows: 0–4 min, 40–80% B; 4–6 min, 80% B; 6–7 min, 80–40% B; 7–8 min, 40% B. The data were acquired in multiple reaction monitoring MRM mode. The sample injection volume was 5 μL. The capillary voltage was 4000 V. The nebulizer was 45 psi. The gas temperature was 340 °C, and the flow rate was 11.0 L/min. MS parameters of 60 compounds were shown in Table S2.

### 2.3. Preparation of Standard Solution

Precisely weighed standards were dissolved in MeOH in 10 mL brown volumetric flasks to prepare stock solutions and stored away from light at −18 °C. The stock solutions were diluted with MeOH to yield working solutions with the desired concentrations and kept at 4 °C.

### 2.4. In-House Fabricated One-Step SPE Cartridge

Self-fabricated rapid filtration cartridges were prepared by sequentially packing sorbents from bottom to top: 300 mg MgSO_4_, 296 mg C_18_, 170 mg PSA, and 35 mg GCB. Each sorbent layer was separated by sintered polyethylene frits, and the polyethylene frits possessed a pore size 20 μm with a thickness of 2.5 mm. A 0.22 μm microporous filter membrane was assembled at the bottom outlet of the cartridge.

### 2.5. Sample Pretreatment

**Figure 1:**
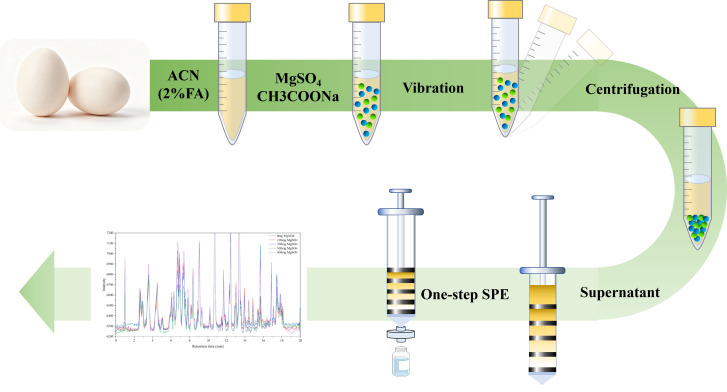
Analysis procedure of sample preparation using developed method.

Briefly, 2.0 g of homogenized egg sample was accurately weighed into a 50 mL polystyrene centrifuge tube with internal standard of chloramphenicol-d_5_, toltrazuril-d_3_ and ponazuril-d_3_ (20 μg/kg), and 10 mL of 2% FA ACN solution was added and mixed thoroughly. The mixture was shaken at 300 rpm for 2 min. Subsequently, 5.0 g of desiccant mixture (anhydrous magnesium sulfate:anhydrous sodium acetate = 4:1, *w*/*w*) was added immediately, followed by vortex mixing, oscillation at 300 rpm for 2 min and then centrifugation at 3000 rpm for 5 min. The supernatant was passed through a self-made SPE cartridge at a flow rate of one drop per second. The collected eluate was evaporated to dryness under nitrogen, followed by reconstitution with 75% MeOH (Figure 1).

### 2.6. Methodological Validation

In line with the EU official guideline SANTE/11312/2021 [21], a comprehensive method of validation covering the limit of detection (LOD), limit of quantification (LOQ), linearity, matrix effect (ME), recovery and relative standard deviation (RSD) was performed under optimized experimental parameters. The LOD was established based on an S/N ratio of 3. The lowest spiked concentration satisfying the method validation criteria (recoveries of 70–120% and RSD ≤ 20%) was defined as the LOQ of each analyte. Meanwhile, all S/N ratios of reported LOQs should ≥ 10. Serial gradient mixed standard working solutions were fortified into blank egg matrices to construct matrix-matched calibration curves covering the concentration range from LOQ to 100× LOQ. Blank egg matrices used in this study were purchased from local supermarkets in Lanzhou, Gansu Province. To confirm that these samples contained no detectable target analytes, blank egg extracts were subjected to the LC-MS/MS analysis procedure described herein. No chromatographic peaks consistent with the retention time and characteristic ion transitions of any target compound were detected in the blank matrices.

ME was calculated as the slope ratio of the matrix-matched calibration curve to the neat solvent calibration curve. Briefly, ME = 1 indicated the absence of matrix interference; ME < 1 corresponded to ion suppression, whereas ME > 1 represented ion enhancement. Based on the classification criteria reported in the reference, ME values from 0.8 to 1.2 were categorized as weak ME. Moderate ME was defined as ME ranging from 0.5 to 0.8 or 1.2 to 1.5; ME below 0.5 or above 1.5 was classified as strong ME [22].

Recovery and precision (RSD) were evaluated at three spiked concentrations (LOQ, 2× LOQ and 10× LOQ), with six parallel replicates prepared for each level. The apparent recoveries were calculated via Equation (1). Both intra-day and inter-day precision were investigated herein: Intra-day precision was expressed as the RSD of six replicates analyzed within a single working day, while inter-day precision was obtained by analyzing identical spiked samples over three consecutive days. The corresponding intra-day and inter-day RSD values were finally computed to assess the repeatability and reproducibility of the proposed method.

(1) $$\mathrm{Recovery}\;(\%)=\frac{\mathrm{Concentration}\;\mathrm{quantified}\;\mathrm{by}\;\mathrm{matrix}-\mathrm{matched}\;\mathrm{calibration}\;\mathrm{curve}}{\mathrm{Theoretical}\;\mathrm{spiked}\;\mathrm{concentration}}\times 100$$

### 2.7. Statistical Analysis

SPSS Statistics software 27.0.1 (IBM Software Inc., Armonk, NY, USA) was used for the paired *t*-tests. *p* < 0.05 was considered statistically significant. A three-factor three-level Box–Behnken design and statistical quadratic model were performed by the Design Expert Version13 (Stat-Ease, Inc., Minneapolis, MN, USA).

## 3. Results and Discussion

### 3.1. Extraction Solution Acidity

**Figure 2:**
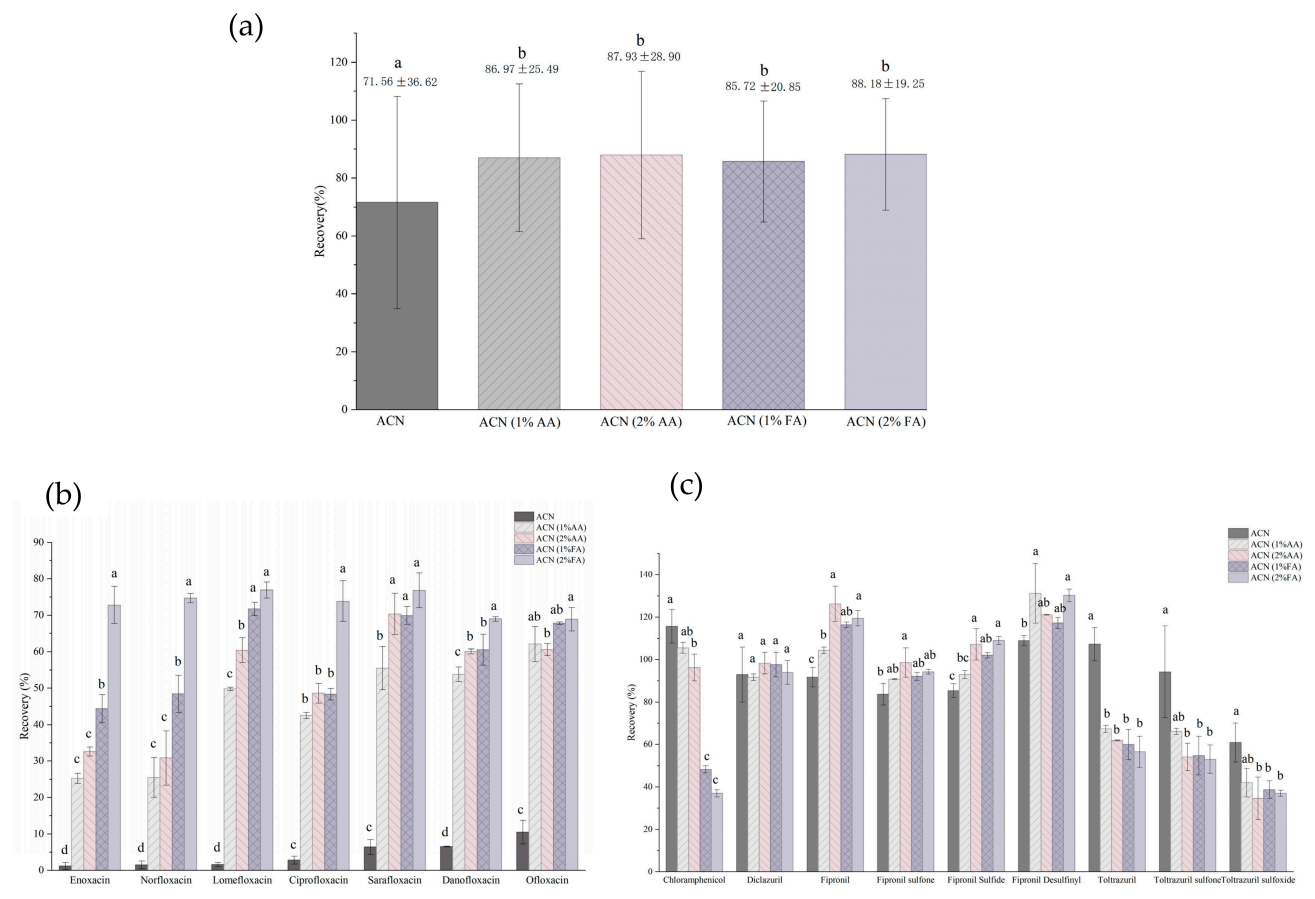
Comparison of recovery on different extraction solution acidity: (**a**) 60 analytes, (**b**) quinolones, (**c**) negative analytes. Note: Different lowercase letters indicate significant differences via Duncan’s test, *p* < 0.05.

Previous studies have verified that ACN is an efficient solvent for simultaneous extraction of multi-pesticide residues, with fewer interfering impurities in extracts and easy liquid–liquid separation with saturated brine [23]. Compared with pure ACN extraction, the addition of AA or FA into acetonitrile improved the overall extraction recoveries of multi-class veterinary drugs in egg matrix [24]. This study compared the recoveries obtained with acetonitrile of different acidities containing ACN, ACN (1% AA), ACN (2% AA), ACN (1% FA) and ACN (2% FA), as shown in Figure 2a. This bar chart compared the average absolute recovery (%) and standard deviation (SD) of target analytes using five systems. Acidification of ACN improves overall recovery to above 85% for all acid-modified groups. ACN (2% FA) shows a slightly higher average recovery of 88.18% with a smaller SD of 19.25 meaning less inter-analyte fluctuation in average recovery. Data on the average recoveries of individual analytes in different extraction solution acidity were summarized in Table S3. The acidity of the extraction solution exerted a particularly prominent effect on quinolone analytes, as shown in Figure 2b. The FA showed superior extraction capacity compared with acetic acid at the same concentration. The solvent of ACN (2% FA) achieved the maximum recoveries for all target quinolones, ranging from 73% to 78%. As shown in Figure 2c, AA and FA exert opposite extraction effects on ESI^−^ detectable compounds. Recovery of the chloramphenicol and toltrazuril series declined in acidified acetonitrile. By contrast, fipronil and its metabolites gain higher recoveries with increasing acid concentration. Diclazuril recovery remains nearly unchanged under all acid conditions. In summary, ACN (2% FA) was selected as the extraction solvent due to satisfactory recoveries for most target analytes. For chloramphenicol and toltrazuril and its metabolites, chloramphenicol-d5, toltrazuril-d_3_ and ponazuril-d_3_ were applied as matched isotopic internal standards to eliminate quantification bias.

In positive-ion mode, for quinolones, neutral zwitterionic forms dominate at near-neutral pH. Raw homogenized egg has a native pH of 6.0–8.5, overlapping with quinolones’ isoelectric zone (Figure 2b), matching the observation reported previously [25]. It will result to severe extraction loss via protein hydrophobic adsorption and metal chelation. Higher extractant acidity fully protonates the analytes’ basic nitrogen groups to cations. Electrostatic repulsion releases protein-bound analytes, while excess H^+^ disrupts metal chelates. Acidic conditions also inhibit quinolone degradation and improve solubility, resulting in continuously elevated recoveries as acidity increases. In negative ESI mode, weak organic acid additives degrade MS response and solution stability for chloramphenicol and toltrazuril metabolites, as low pH primarily suppresses analyte deprotonation. However, these acids enhance signals of fipronil derivatives, matching the published results of Wu et al. [26]. Reduction reactions contribute to ion generation under negative electrospray. Acid modifiers supply extra protons to accelerate reduction, allowing spray droplets to accumulate excess negative charge at the droplet surface via electrostatic repulsion. The elevated local surface pH facilitates deprotonation of neutral halogenated fipronil molecules, overriding the inhibitory effect of acidic solvent.

### 3.2. Purification

C_18_, PSA, GCB and MgSO_4_ are commonly used purification materials for extraction of complex matrices [27,28]. Most previous studies optimized the dosage of cleanup sorbents solely via single-factor experiments [28,29,30,31,32]. In this work, single-factor analysis was first conducted to screen critical variables, followed by RSM. This two-step strategy highlights the unique advantages of response surface analysis which accurately locates the global optimal combination of sorbent dosages rather than a local optimum [33]. The single-factor experiments were carried out to screen the optimal dosage of each SPE sorbent separately. The performance was evaluated based on the count of analytes satisfying the validation criteria (recovery 70–120%, RSD < 20%). The optimum dosage of each sorbent obtained from single-factor tests was adopted as the central level of three-factor response surface analysis. A Box–Behnken design with three levels for each variable was employed to explore the quadratic effect of individual sorbent dosage and the interactive effects between different cleanup materials. Analysis of variance was utilized to identify significant linear, quadratic and cross-interaction terms, and contour and 3D response surface plots were generated to interpret synergistic or antagonistic interactions among C_18_, PSA, GCB and MgSO_4_.

#### 3.2.1. Single-Factor Experiment

**Figure 3:**
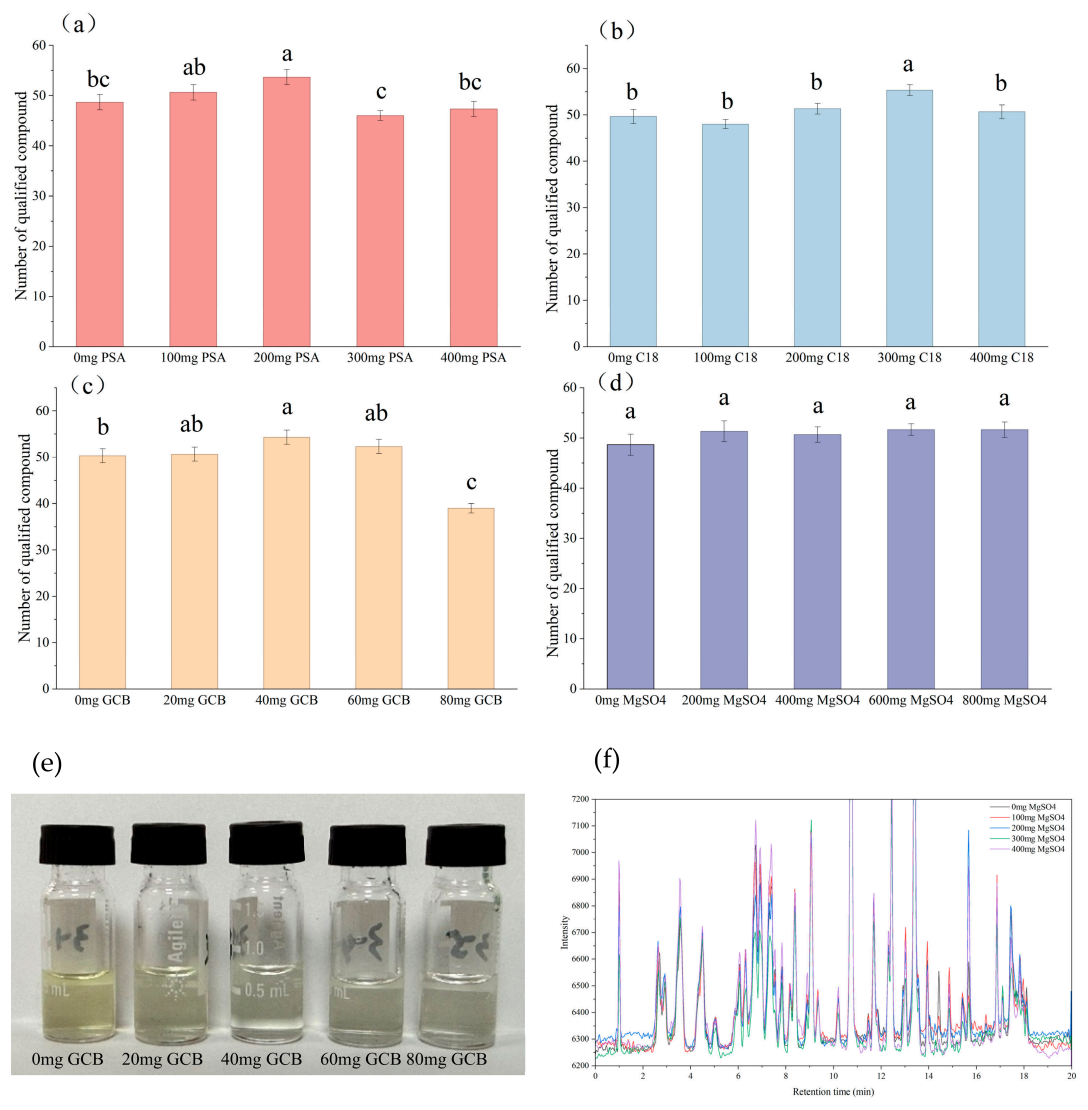
Single-factor optimization of sorbent dosages for egg sample pretreatment. (**a**) Bar charts of qualified target compound numbers under different PSA. (**b**) Bar charts of qualified target compound numbers under different C_18_. (**c**) Bar charts of qualified target compound numbers under different GCB. (**d**) Bar charts of qualified target compound numbers under different MgSO_4_. (**e**) Photographs of egg extracts with increasing GCB. (**f**) TIC of baseline noise variation in extracts treated with graded C_18_ amounts. Note: Different lowercase letters indicate significant differences via Duncan’s test, *p* < 0.05.

Single-factor experiments were carried out to individually optimize the dosage of four SPE sorbents (PSA, C_18_, MgSO_4_ and GCB) for multi-class residue detection in egg matrix. The number of target analytes satisfying the method validation criteria (recoveries of 70–120% and RSD < 20%) was selected as the core evaluation indicator, and Duncan’s multiple range test was adopted to identify statistical differences between dosage groups, where distinct lowercase letters above bars represent significant differences at *p* < 0.05 (Figure 3). The error bars represent standard deviation.

PSA sorbent primarily interacts with matrix interferents via hydrogen bonding. This mechanism enables the removal of co-extracted impurities such as fatty acids, organic acids, and partial sugars and pigments [29]. For the PSA sorbent shown in Figure 3a, the maximum count of qualified compounds was obtained at 200 mg, which was significantly superior to the groups of 0, 300 and 400 mg. Excessive PSA retained polar and amphiprotic analytes such as sulfonamides and quinolones via amino groups, causing substantial loss of validated targets. Therefore, 200 mg was selected as the central level for subsequent response surface design.

C_18_ removes lipid co-extractives via hydrophobic interaction between octadecyl chains and triglycerides in egg yolk [30]. The 300 mg C_18_ treatment yielded a significantly higher count of qualified compounds compared with the 0, 100, 200 and 400 mg groups (Figure 3b). No improvement was observed at 400 mg, as the adsorption capacity of C_18_ reached saturation and only a trivial loss of highly hydrophobic pesticides occurred. Accordingly, 300 mg was selected as the central level for subsequent response surface design.

GCB is a sorbent which can adsorb non-polar pigments and sterols [30]. The maximum count of qualified compounds was obtained at 40 mg GCB, which was superior to the 0 mg and 80 mg groups (Figure 3c). The photographs of extracts visually illustrated the decolorization effect of GCB are shown in Figure 3e: The raw extract without GCB displayed obvious yellow pigmentation originating from lutein and zeaxanthin, and the solution turned transparent with increasing GCB addition up to 40 mg via π-π stacking adsorption of planar carotenoid impurities. However, excessive GCB at 80 mg induced a sharp decline in validated analytes. The non-selective π-π interaction of GCB simultaneously retained planar conjugated target compounds including quinolone and aflatoxins, leading to severe loss of recoveries beyond the acceptable range, as previously reported [31,34]. Considering both statistical performance and visual decolorization efficiency, 40 mg was selected as the central level for subsequent response surface design.

MgSO_4_ removes residual water from the extraction system. Dehydration elevates the ionic strength of the organic phase, which further improves extraction recovery of target analytes [32]. Duncan’s multiple range test revealed no statistically significant differences across all MgSO_4_ treatments (0–800 mg), as shown in Figure 3d. A loading of 200 mg was sufficient to thoroughly eliminate aqueous interferences in acetonitrile extracts. Higher MgSO_4_ levels only slightly increased ionic strength and induced trivial co-precipitation of hydrophobic analytes, which failed to produce observable variation in the count of qualified compounds. Furthermore, total ion chromatograms (TICs) were compared, as shown in Figure 3f, extracts without MgSO_4_ exhibited elevated baseline noise derived from residual water-soluble matrix components, while consistent low-baseline profiles were obtained once MgSO_4_ dosage exceeded 200 mg. Therefore, 300 mg was selected directly without subsequent response surface design.

#### 3.2.2. Response Surface Methodology

Numerical optimization was carried out on the established quadratic regression model to maximize the number of validated target analytes within the dosage ranges of PSA (100–300 mg), GCB (20–60 mg) and C18 (200–400 mg). A three-factor three-level Box–Behnken design was performed with PSA, GCB and C_18_ as independent variables, as shown in Table S4a, and the number of target analytes meeting validation criteria was selected as the response value to establish a second-order polynomial regression model. The overall model was highly significant (F = 34.69, *p* < 0.0001), and the lack-of-fit term was insignificant (*p* = 0.1731), indicating no systematic deviation between the regression model and experimental data (Table S4b). As shown in Equation (2) and Table S4b, linear terms PSA and GCB exerted significant negative effects on the response, while C18 showed an significant positive influence. All pairwise interaction terms (AB, AC, BC) were insignificant, revealing no obvious synergistic or antagonistic interactions among the three cleanup sorbents. The quadratic terms A^2^, B^2^ and C^2^ were all significant with negative coefficients, forming downward-opening parabolic surfaces with an optimal peak for each sorbent dosage. The model exhibited excellent fitting performance with R^2^ = 0.9781, adjusted R^2^ = 0.9499 and Predicted R^2^ = 0.7514, where the gap between adjusted and predicted R^2^ was less than 0.2. The low coefficient of variation (C.V. = 3.47%) and adequate precision of 16.39 demonstrated favorable repeatability and reliable prediction capacity (Table S4c), which could be applied to solve the globally optimal combined sorbent formulation for simultaneous multi-class residue analysis. The globally optimal cleanup formulation with a desirability of 1.000 was determined as 170 mg PSA, 35 mg GCB and 296 mg C18, with a predicted quality value of 60.02. A series of verification experiments (n = 5) were then carried out to validate the consistency of the actual recoveries and the theoretical predictions under the optimum condition. The result showed that deviations between the obtained results and the predicted values were less than 10%, which indicated that the prediction accuracy of the established model was acceptable.

**Figure 4:**
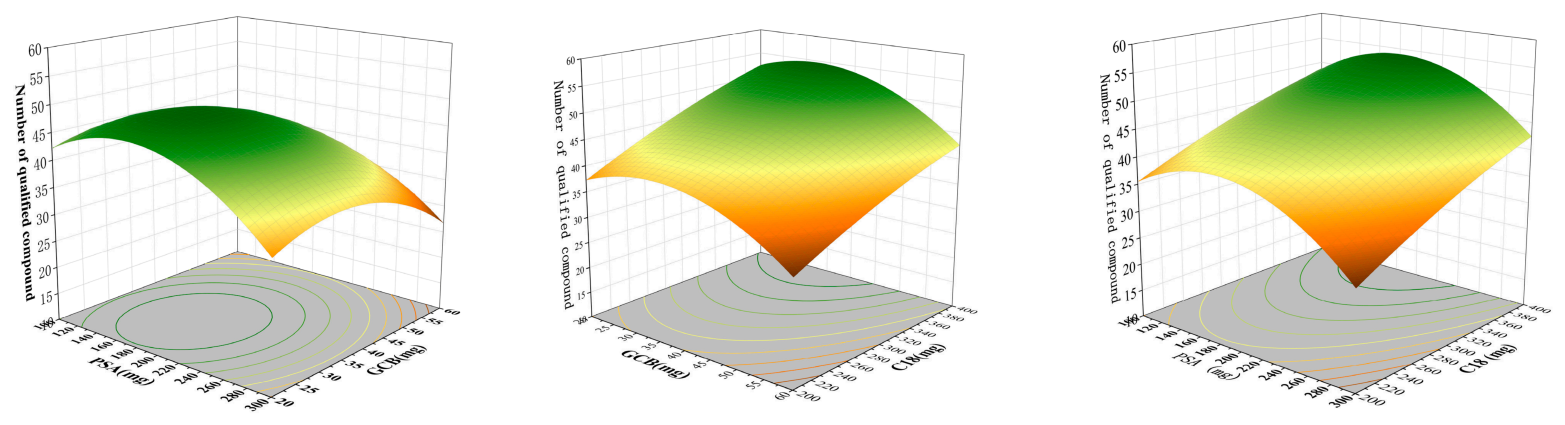
The 3D response surface of qualitied analytes by PSA, GCB and C_18_.

The relationship between qualified compounds and the three selected components is represented by in Equation (2), as suggested by the statistical quadratic model from the Design Expert Version 13. The 3D response surface was showed in Figure 4.

(2) Y = −22.25 + 0.25975A + 1.01125B + 0.140750C + 0.0005AB + 0.000038AC − 0.000312BC − 0.000815A^2^ − 0.015375B^2^ − 0.00011C^2^

where Y represented qualified compounds; and A, B and C were the coded variables for the amount of PSA, GCB and C18, respectively.

### 3.3. Optimization of Diluents

The diluent of the injection solvent is a critical, yet often underestimated, parameter in LC-MS/MS. The previous studies acknowledge solvent effects; they lack systematic evaluation across polarity groups and fail to establish predictive rules for solvent optimization [35]. MS response was greater in MeOH than ACN for most analytes, which was attributed tomethanol’s protic nature and its ability to solvate lone pairs [36]. In this study, MS response of four diluents (25% MeOH, 50% MeOH, 75% MeOH and MeOH) were compared to establish rules for solvent optimization.

**Figure 5:**
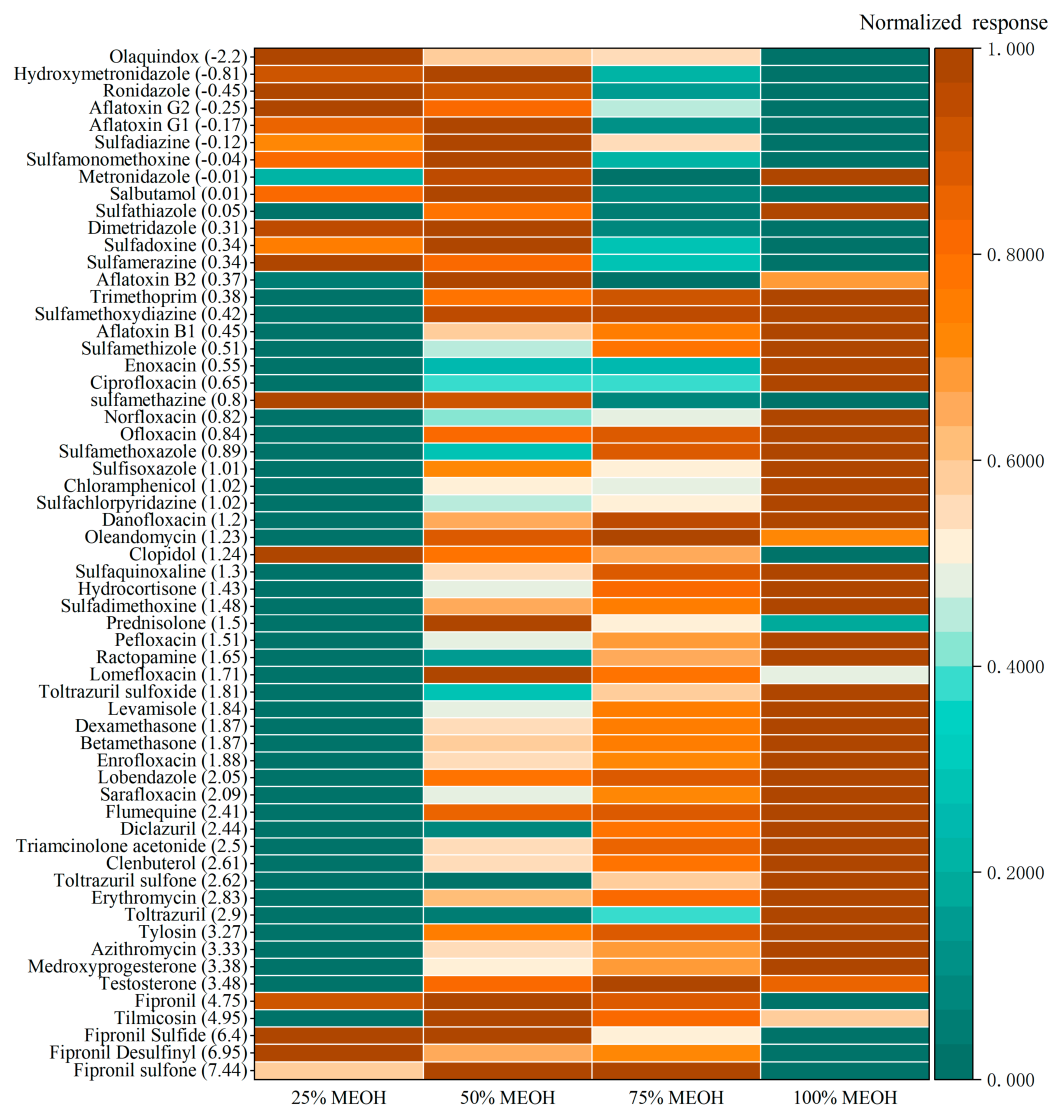
Heatmap of the MS response of 60 analytes. Note: Numbers in brackets denote log P for individual analytes.

For highly polar analytes with negative log P at the top of Figure 5, the normalized response decreased continuously as MeOH proportion increased, with the maximum signal achieved at 25% MeOH. Those compounds (olaquindox, metronidazole, aflatoxins, etc.) are detected under ESI^+^ mode via protonation to generate [M+H]^+^, whose MS response is affected by proton availability and aqueous solubility. At 25% MeOH with an abundant water fraction, hydrogen-bonded water networks efficiently solvate and transport free protons, facilitating sufficient proton capture by polar molecules distributed in the aqueous core of charged electrospray droplets, which produces the maximum ionization efficiency. The present findings corroborate the conclusions from Eddahby, who proposed that for these polar analytes, higher water content elevates surface tension, promotes more efficient droplet fission and desolvation, and thereby facilitates fuller gas-phase ion generation of polar, easily ionizable analytes [37]. As the MeOH proportion increases, a high organic fraction lowers solvent polarity, slightly reducing the solubility and dispersion of these hydrophilic compounds. In addition, the loss of water weakens proton transfer capacity, while excess MeOH competes for free protons and reduces the effective proton concentration available for analytes. Combined insufficient proton supply and poor molecular dispersion continuously suppress the formation of [M+H] ^+^, resulting in a monotonic decline of normalized response from 25% to 100% MeOH.

Amphoteric veterinary drugs with moderate log *p* values (0–4.5), including fluoroquinolones, sulfonamides, macrolides and glucocorticoids, generate [M+H]^+^ under ESI^+^ mode and exhibit a monotonic increase in normalized MS response with rising MeOH fraction, featuring a response valley at 25% MeOH and the maximum signal at 100% pure MeOH. At 25% MeOH dominated by water, the aqueous solvent provides insufficient free protons for protonation of alkaline amino groups. Meanwhile, the hydrophobic skeletons of amphoteric analytes are excluded from the water-rich droplet surface, trapping most target molecules inside the aqueous core and preventing them from accessing the electric field for ionization; additionally, strong hydrogen bonding between analytes and water triggers molecular aggregation, further reducing the concentration of ionizable monomers. As MeOH proportion increases, the protic organic solvent continuously elevates total free hydrogen ion concentration, and surface-active MeOH gradually replaces water at the droplet-air interface, allowing hydrophobic segments of amphoteric molecules to anchor at the ionization zone [36]. In 100% MeOH, the system achieves the highest proton abundance, elimination of droplet partitioning exclusion, and negligible analyte aggregation. Although the absence of water weakens proton transportation via hydrogen-bond networks, the ultrahigh concentration of free protons fully compensates for this disadvantage, resulting in optimal protonation efficiency and the strongest MS signal among all tested dilution solvents.

Fipronil and its metabolites have log *p* values greater than 4.5, which exhibited the highest response in 50% MeOH. Theoretically, they show the highest solubility in pure MeOH and are supposed to yield the maximum MS response. Nevertheless, these compounds are analyzed in negative ESI^−^ mode via deprotonation to form [M-H]^−^. MeOH acts as a proton donor through self-dissociation; systems with a high MeOH fraction release abundant free H^+^. In accordance with Le Chatelier’s principle, elevated proton concentration suppresses the dehydrogenation of target analytes and hinders the production of anions, resulting in the lowest response for the 100% pure MeOH group [36]. As the MeOH proportion decreases from 100% to 50%, the increased aqueous fraction dilutes free protons dissociated from MeOH and alleviates dehydrogenation inhibition, leading to a gradual rise in MS response. When the MeOH fraction is further reduced to 25%, the excessively high water content reduces the solubility of highly hydrophobic fipronil analogs, causing a decline in signal response. Therefore, the highest MS response exhibited in 50% MeOH.

Comprehensively considering the polarity and ionization efficiency of all target analytes under the corresponding ionization modes, 75% methanol was selected as the diluent. Although it could not deliver the maximum MS signal intensity for individual compounds, it achieved balanced responses for analytes with wide-ranged polarities. Consistent with previous research, the 75% MeOH exhibited optimal analytical performance, generating higher, more reproducible peak areas [35].

### 3.4. Methodology Validation

#### 3.4.1. Linear Range LOD and LOQ

The linearity, correlation coefficient, LOD and LOQ of the developed LC-MS/MS method for 60 target analytes in egg matrix are summarized in Table 1. Matrix-matched calibration curves were applied to eliminate matrix suppression effect, and all analytes exhibited favorable linear responses within corresponding concentration ranges. The correlation coefficients (R^2^) of all regression equations were higher than 0.9916, and more than 90% of compounds achieved R^2^ > 0.9990, demonstrating excellent linearity. The LODs of all targets were 0.1–3 μg/kg. The LOQs of all targets ranged from 0.2 to 10 μg/kg, where fipronil sulfone, flumequine, most sulfonamides and β2-agonists presented ultra-low LOQs of 0.2 μg/kg, which fully satisfied the residual monitoring requirements of national food safety standards (GB 31658, GB 31659 series) for both restricted and prohibited veterinary drugs in eggs.

#### 3.4.2. Matrix Effect

ME was calculated by comparing the slope of matrix-matched calibration curve with solvent standard curve, and ME values of all 60 analytes were listed in Table 1. According to the evaluation criterion, ME ranging from 0.8 to 1.2 represented negligible matrix interference, which covered more than 60% target compounds including aflatoxins, most sulfonamides, nitroimidazoles and fluoroquinolones. The optimized SPE cleanup procedure largely reduced lipid and pigment interferences, and minimized the deviation of ME values for most veterinary drugs.

The severe matrix enhancement (ME = 3.00–3.17) of fipronil and its metabolites in egg matrix electrospray negative ion mode originated from their planar conjugated hydrophobic molecular structure and abundant lipid co-extractives in yolk. Fipronil derivatives possess rigid aromatic π-conjugated skeletons with strong electron-withdrawing trifluoromethyl and cyano groups, which readily form stable deprotonated anions [M-H]^−^. Residual triglycerides and phospholipids after SPE cleanup act as ionization promoters: long-chain lipid anions enriched on the surface of ESI droplets facilitate proton transfer and moderately improve the deprotonation efficiency of fipronil. Therefore, fipronil and its metabolites showed severe matrix enhancement. Erythromycin (ME = 0.14) and olaquindox (ME = 0.27) exhibited serious matrix suppression. Erythromycin, with multiple hydroxyl groups and a basic dimethylamino group, is a basic macrolide that readily forms electrostatic and hydrogen-bond complexes with egg phospholipids and soluble proteins. Olaquindox is a polar quinoxaline-N, N-dioxide compound with strong electron-withdrawing nitro and N-oxide functional groups, which are co-eluted with egg lipoproteins and saccharides. These co-extracted matrix impurities compete for proton charge in the ESI source and lead to severe ionization suppression for both analytes. To mitigate the strong matrix effects of individual analytes, a matrix-matching standard curve was necessary for quantitative analysis to obtain reliable results.

#### 3.4.3. Accuracy and Precision

The accuracy and precision of the established multi-residue LC-MS/MS method were validated at three spiked levels (1× LOQ, 2× LOQ and 10× LOQ) in blank egg matrix. Intra-day precision was determined with six parallel replicates on a single day, while inter-day precision was evaluated over three consecutive working days. The mean recoveries of all 60 target analytes ranged from 70.19% to 119.06%, fully satisfying the acceptable recovery range of 70–120%. The intra-day RSDs of all compounds fell between 0.09% and 19.64%, and inter-day RSDs varied from 2.05% to 19.30%. All RSD values were below the critical limit of 20%, demonstrating satisfactory repeatability and reproducibility. Inter-day RSD values were generally slightly higher than intra-day RSDs, which could be attributed to minor fluctuations in matrix background and mass spectrometric instrument status across different experimental days. Nevertheless, all deviations remained within acceptable limits, verifying favorable laboratory reproducibility of the entire pretreatment workflow.

### 3.5. Compared Results

**Figure 6:**
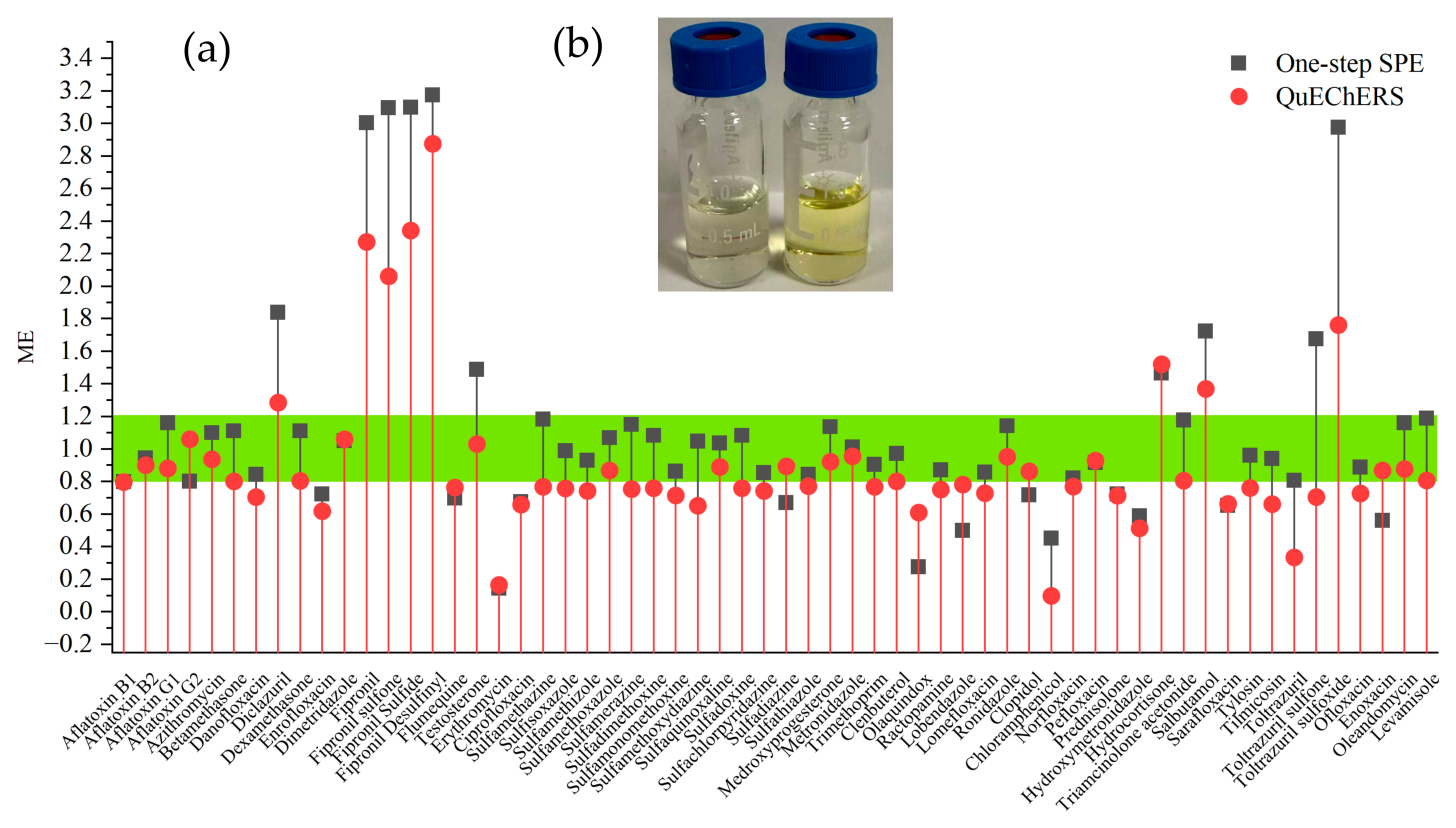
Comparison of one-step SPE method and QuEChERS. (**a**) ME; (**b**) color of purified extract. The green shaded region corresponds to the weak ME range of 0.8–1.2.

The comprehensive performance of the self-optimized PSA-GCB-C_18_-MgSO_4_ mixed one-step SPE method was compared with the commercial Agilent EMR-Lipid QuEChERS kit, and the proposed method displayed practical advantages. The whole pretreatment workflow took only 20 min per sample, one-third shorter than the 30 min operation of EMR-Lipid QuEChERS, and the simplified SPE steps largely improved throughput for routine batch screening of egg samples. Meanwhile, the mixed sorbent only cost ¥10 for every single test, merely 1/5 of the commercial EMR-Lipid kit priced at ¥50 per sample, which greatly cut long-term laboratory expenditure in large-scale residue monitoring. Experimental results verified that the sorption of C_18_, GCB and PSA simultaneously eliminated lipids, carotenoid pigments and free fatty acids from egg yolk, enabling weak ME (0.8–1.2) for a total of 35 target analytes, far more than the 21 analytes obtained via EMR-Lipid treatment and effectively mitigating ion suppression or enhancement for various analytes categories (Figure 6a). Benefiting from GCB, the final purified extract was fully colorless and transparent, whereas pale yellow pigment residue remained in EMR-Lipid extracts without carbon black additive (Figure 6b); continuous injection of colorless extracts alleviated cumulative contamination on LC columns and ESI ion sources and reduced instrument maintenance frequency. It should be noted that the EMR-Lipid sorbent possessed superiority targeting fipronil and its metabolites. Based on size-exclusion selective adsorption, EMR-Lipid specifically removed phospholipids and triglycerides [34,38,39]—the primary contributor to severe ion enhancement of fipronil—thus delivering lower ME values of the fipronil series (2.1–2.8) than our mixed sorbent method (3.0–3.2). However, this advantage only worked for fipronil derivatives. Considering the comprehensive demands of simultaneous multi-residue detection covering purification performance, analysis speed and economic efficiency, the mixed-sorbent method developed in this study achieved wider applicability and better practical value for egg matrix monitoring.

The performance of the proposed method for multi-contaminant analysis in eggs was compared with previously reported approaches adopting diverse cleanup strategies (Table 2). The LOQ range of 0.2–10 μg/kg obtained in the present study is comparable to, or even superior to, those documented in existing studies. The recovery falls within the internationally recognized validation thresholds for multi-residue analytical methods, demonstrating favorable analytical accuracy and precision. More importantly, the proposed pretreatment workflow features simplified operational procedures and reduced experimental expenditure. The self-packable SPE cartridges eliminate reliance on commercial purification kits, rendering this methodology highly applicable to high-throughput routine screening of large batches of egg samples in analytical laboratories.

### 3.6. Evaluation of Greenness of the Developed Method

Against the backdrop of strengthened national environmental governance and rising public ecological awareness, quantitative green assessment of analytical pretreatment methods has gained wide research interest. Two standardized evaluation tools, AGREEprep and the Blue Applicability Grade Index (BAGI), are used to separately assess the environmental friendliness and laboratory practicality of analytical workflows. AGREEprep evaluates pretreatment greenness via twelve environmental criteria, while BAGI assesses operational practicability from ten practical dimensions.

**Figure 7:**
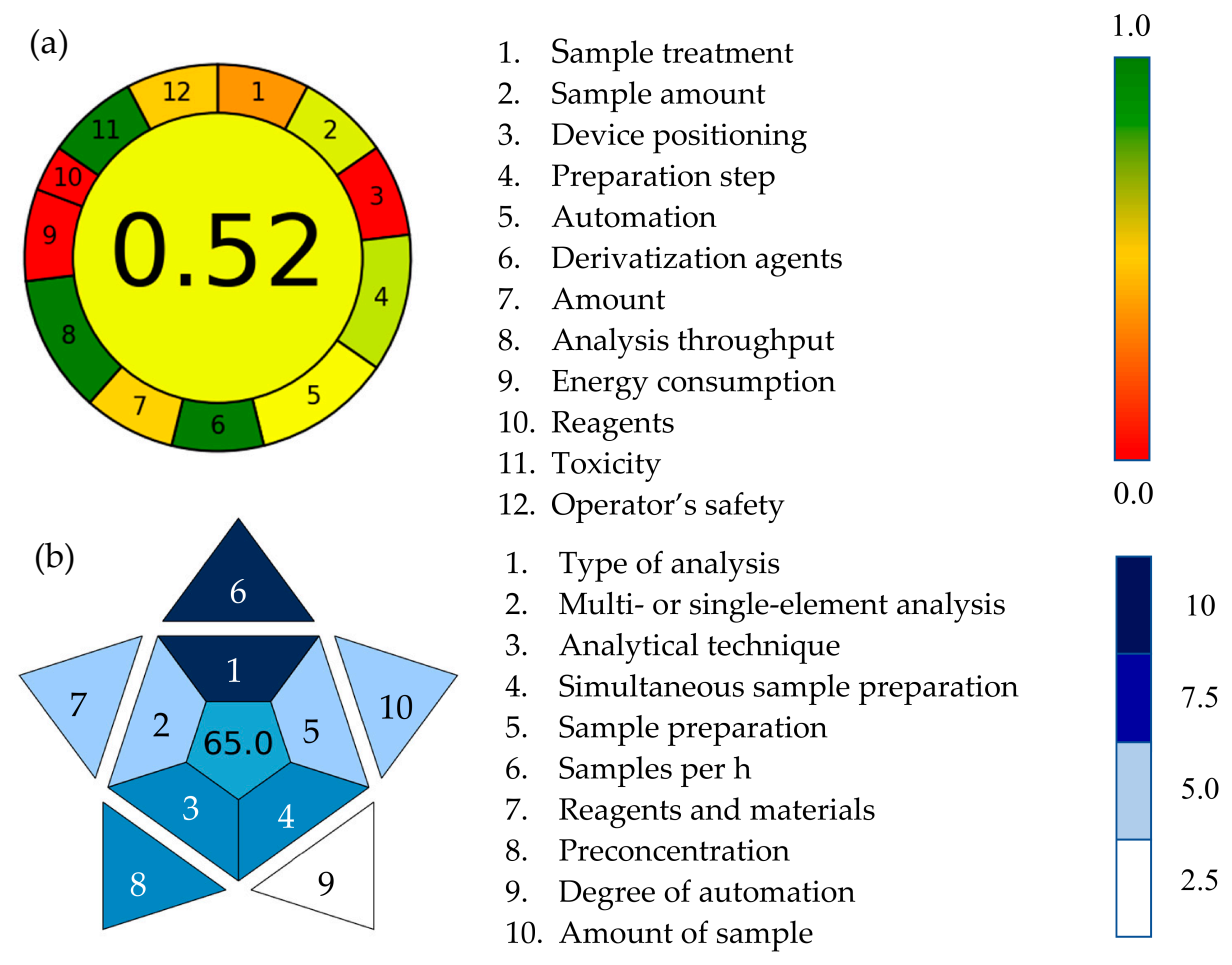
The greenness score of the developed method evaluated by (**a**) AGREEprep method and (**b**) BAGI method.

As shown in Figure 7a, the AGREEprep overall score of the developed homemade one-step SPE method was 0.52 (0–1 scale), representing moderate green performance. Optimization space exists for analytical device, energy consumption and operator safety, though the method exhibits favorable green performance in sample throughput and toxic reagent use.

The BAGI evaluation (Figure 7b) yielded a qualified practicality score of 65.0 (25–100 scale), proving its suitability for routine egg veterinary drug multi-residue screening. The workflow boasts prominent practical merits, including simultaneous multi-analyte detection, integrated extraction-cleanup, microscale sample consumption and universal LC-MS/MS compatibility. However, low automation capacity is the main drawback, as full manual operation restricts unattended high-throughput batch testing.

### 3.7. Real Samples

**Figure 8:**
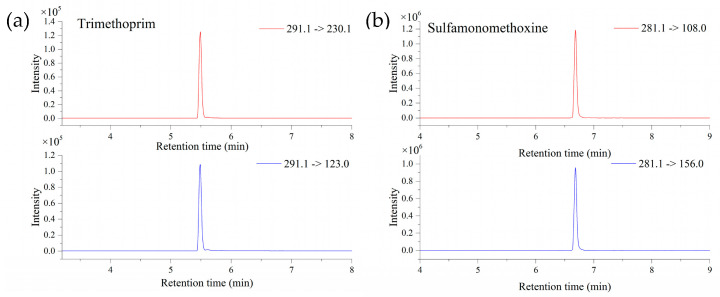
Chromatograms of authentic positive samples. (**a**) Trimethoprim. (**b**) Sulfamonomethoxine.

The developed analytical method was applied to analyze 100 batches of fresh egg samples. Sulfamonomethoxine and trimethoprim were simultaneously detected in one positive batch, with measured concentrations of 1396 μg/kg and 409 μg/kg, respectively (Figure 8). In accordance with GB 31650.1-2022 National Food Safety Standard—Maximum Residue Limits of 41 Veterinary Drugs in Food [43], the maximum residue limit (MRL) for total sulfonamides and trimethoprim in eggs is set at 10 μg/kg. Consequently, this sample was determined to be non-compliant with the regulatory limits. The extremely high residues of sulfamonomethoxine and trimethoprim detected in this single sample can be attributed to non-compliant breeding practices, or the failure to observe the legally mandated withdrawal period prior to egg collection. Corresponding regulatory intervention measures have been implemented by local market supervision authorities to prevent similarly non-compliant eggs from re-entering the market circulation chain.

Subsequent confirmatory testing of the same sample was performed using two official standard methods: Announcement No. 1025-23-2008 issued by the Ministry of Agriculture of China [44] and SN/T 2538-2010 [45]. The measured concentrations of sulfamonomethoxine and trimethoprim obtained from the standard methods were 1213 μg/kg and 458 μg/kg. The relative deviation between the results of the self-established method and the official standard methods was less than 20%, which further verified the accuracy and reliability of the proposed method.

## 4. Conclusions

In this study, an in-house fabricated one-step SPE cartridge combined with HPLC-MS/MS was developed for the simultaneous quantitative analysis of 60 multi-class contaminants, including veterinary drugs, pesticides and mycotoxins, in egg samples. Response surface methodology was applied to systematically optimize the sorbent formulation of the self-packed SPE column. This one-step cleanup protocol enables selective retention of egg matrix interferences such as lipids, proteins and pigments by the sorbent during sample loading, while all 60 target veterinary drugs, pesticides and mycotoxins pass through the sorbent directly into the eluate. Satisfactory linearity (R^2^ > 0.99), low LOQs ranging from 0.2 to 10 μg/kg, acceptable recoveries (70–120%) and good precision (RSD < 20%) were obtained for all target analytes with broad log *p* values from −2.2 to 7.4. Weak matrix effects were observed for most compounds after SPE cleanup, demonstrating prominent matrix interference removal performance toward lipid-rich egg yolk matrices. Compared with QuEChERS (EMR-Lipid), the proposed method features a more streamlined experimental workflow, shorter pretreatment duration, weaker matrix effects, and substantially lower analytical costs. Furthermore, the established method was successfully applied to real commercial egg sample screening; sulfamonomethoxine and trimethoprim were detected in one batch of eggs. It was verified by the official standard methods, showing reliable practicability for routine food safety surveillance. In summary, this integrated one-step SPE-HPLC-MS/MS approach provides a universal, efficient and sensitive technical tool for synchronous monitoring of combined chemical contamination in eggs, and holds great potential for risk assessment of animal-derived food products.

## Figures and Tables

**Table 1:** Methodology validation parameters of 60 analytes.

Compounds	Linearity Range (μg/kg)	Correlation Coefficient	Regression Equation	LOD ^a^ (μg/kg)	LOQ ^b^ (μg/kg)	ME ^c^	Intra-Day Precision (n = 6) and Recovery (%)						Inter-Day Precision (n = 18) (%)		
							1× LOQ REC ^d^	1× LOQ RSD ^e^	2× LOQ REC ^d^	2× LOQ RSD ^e^	10× LOQ REC ^d^	10× LOQ RSD ^e^	1× LOQ RSD ^e^	2× LOQ RSD ^e^	10× LOQ RSD ^e^
Aflatoxin B1	0.5–50	0.9998	y = 337.39x − 31.50	0.2	0.5	0.8	72.94	10.78	92.79	4.25	89.03	0.21	19.3	8.3	6.9
Aflatoxin B2	0.5–50	0.9998	y = 219.27x + 1.54	0.2	0.5	0.94	96.02	8.91	70.73	7.45	92.27	10.04	13.03	11.29	8.6
Aflatoxin G1	0.5–50	0.9999	y = 312.52x + 50.98	0.2	0.5	1.16	94.83	14.84	82.04	6.52	89.05	5.43	7.11	7.44	7.18
Aflatoxin G2	1–100	0.9994	y = 81.36x + 23.38	0.3	1	0.8	83.19	4.91	78.75	9.67	73.25	6.4	11.05	4.3	4.77
Azithromycin	2–200	0.9991	y = 46.74x − 24.46	0.7	2	1.1	89.16	11.66	91.13	17.59	78.73	13.71	6.21	7.61	10.95
Betamethasone	0.5–50	0.9999	y = 333.89x + 50.13	0.2	0.5	1.11	119.06	10.13	97.07	2.66	101.77	6.2	10.5	16.75	7.9
Chloramphenicol	2–200	0.9989	y = 22.48x + 45.14	0.7	2	0.45	111.94	14.46	106.3	16.93	90.22	7.56	11.03	11.95	9.53
Ciprofloxacin	1–100	0.9973	y = 398.23x − 168.32	0.3	1	0.67	86.82	1.81	113.4	4.16	104.7	17.08	11.04	10.46	11.24
Clenbuterol	0.2–20	0.9999	y = 510.14x + 104.19	0.1	0.2	0.97	112.06	12.76	98.13	12.17	87.62	4.4	16.33	5.01	14.11
Clopidol	0.5–50	0.9996	y = 187.78x + 6.21	0.2	0.5	0.72	77.1	7.64	99.7	4.26	115.03	2.87	11.26	8.86	3.4
Danofloxacin	5–500	0.9949	y = 243.07x − 392.44	2	5	0.84	87.13	13.36	89.41	10.62	78.68	12.71	12.31	14.86	12.77
Dexamethasone	0.5–50	0.9999	y = 333.89x + 50.13	0.2	0.5	1.11	94.62	10.31	104.26	4.48	96.52	1.42	17.52	5.31	8.51
Diclazuril	2–200	0.9958	y = 36.98x + 160.26	0.7	2	1.84	98.28	12.88	82.29	12.8	105.29	9.61	18.78	14.1	14.46
Dimetridazole	1–100	0.996	y = 44.61x + 30.46	0.3	1	1.05	101.62	9.35	79.57	7.88	87.55	10.61	17.79	5.48	15.18
Enoxacin	0.2–20	0.9982	y = 966.57x − 376.61	0.1	0.2	0.56	116.33	5.84	94.08	7.91	80.19	9.39	18.3	18.7	7.9
Enrofloxacin	1–100	0.9986	y = 352.87x − 361.94	0.3	1	0.72	75.81	5.51	97.97	13.09	90.44	7.55	9.26	9.86	8.43
Erythromycin	0.2–20	0.9999	y = 510.30x − 29.41	0.1	0.2	0.14	111.43	8.62	79.58	7.43	75.92	4.28	16.04	15.92	12.03
Fipronil	0.5–50	0.999	y = 464.10x + 439.79	0.2	0.5	3	101.88	8.41	89.58	15.78	103.91	5.29	5.28	3.64	8.36
Fipronil Sulfide	0.5–50	0.9955	y = 334.86x + 779.68	0.2	0.5	3.1	119.62	18.76	103.42	15.47	93.42	13.44	18.99	12.89	7.29
Fipronil sulfone	0.2–20	0.999	y = 4209.21x + 2854.36	0.1	0.2	3.09	86.02	10.06	76.23	16.93	90.64	16.54	17.96	13.53	17.32
Fipronil Desulfinyl	0.5–50	0.9994	y = 681.95x + 199.51	0.2	0.5	3.17	88.74	10.89	107.61	16.5	95.63	17.8	18.74	13.05	17.01
Flumequine	0.2–20	0.9999	y = 5245.11x + 101.40	0.1	0.2	0.7	100.02	1.03	77.4	8.42	109.69	1.99	11.88	9.92	2.75
Hydrocortisone	2–200	0.9977	y = 29.75x − 5.70	0.7	2	1.46	95.61	5.6	83.69	1.32	107.9	2.04	11.76	6.68	2.88
Hydroxymetronidazole	5–500	0.9972	y = 35.36x − 22.84	2	5	0.59	95.64	10.35	108.68	2.45	97.15	8.39	15.77	12.6	4.78
Levamisole	0.5–50	0.9999	y = 376.17x + 13.42	0.2	0.5	1.19	78.49	10.61	106.25	11.18	93.75	14.58	15.38	2.05	8.86
Lobendazole	1–100	0.9982	y = 254.73x − 61.35	0.3	1	0.5	94.95	5.9	109.83	3.79	105.27	3.16	10.8	15.01	7.35
Lomefloxacin	2–200	0.9916	y = 518.14x − 927.32	0.7	2	0.86	116.84	16.42	99.71	6.01	120.49	8.38	15.11	14.63	6.43
Medroxyprogesterone	0.5–50	0.9996	y = 334.42x + 39.81	0.2	0.5	1.14	119.3	11.29	89.18	17.61	74.1	4.32	7.48	4.73	5.79
Metronidazole	0.5–50	0.9999	y = 245.71x + 19.22	0.2	0.5	1.01	99.95	10.83	102.53	10.11	105.8	9.94	17.95	11.38	3.02
Norfloxacin	1–100	0.998	y = 410.44x − 179.32	0.3	1	0.82	107.65	11.23	81.01	5.08	90.52	3.99	12.54	16.85	15.51
Ofloxacin	2–200	0.9963	y = 450.88x − 652.49	0.7	2	0.89	103.88	6.66	107.83	4.63	104.75	6.25	16.01	16.29	4.11
Olaquindox	1–100	0.9982	y = 134.99x + 19.38	0.3	1	0.27	74.19	12.58	95.52	7.71	118.56	6.26	10.07	12.02	16.86
Oleandomycin	0.2–20	0.9999	y = 741.91x − 84.30	0.1	0.2	1.16	79.86	3.4	78.81	8.56	105.09	14.45	14.89	7.92	15.41
Pefloxacin	2–200	0.998	y = 477.54x − 398.35	0.7	2	0.92	102.32	11.97	86.07	14.01	105.32	6.77	12.7	4.61	13.1
Prednisolone	2–200	0.9993	y = 115.32x − 14.73	0.7	2	0.72	89.03	14.44	74.21	9.2	95.28	16.31	6.01	10.42	4.44
Ractopamine	0.5–50	0.9999	y = 451.05x + 73.09	0.2	0.5	0.87	117.12	4.75	78.28	6.02	84.31	1.16	15.19	10.37	17.05
Ronidazole	0.5–50	0.9997	y = 455.14x + 181.63	0.2	0.5	1.14	73	2.36	119.27	5.24	85.09	2.04	10.17	11	6.55
Salbutamol	0.2–20	0.999	y = 915.38x + 122.72	0.1	0.2	1.72	70.79	10.6	106.88	15.28	110.18	2.35	18.58	11.85	12.42
Sarafloxacin	0.5–50	0.9998	y = 406.32x − 171.02	0.2	0.5	0.65	110.09	4.17	105.03	10.28	103.48	12.43	14.81	6.91	9.13
Sulfachlorpyridazine	1–100	0.9998	y = 187.87x − 11.25	0.3	1	0.85	74.65	11.82	74.07	11.98	79.36	13.46	14.77	10.27	5.05
Sulfadiazine	0.5–50	0.9995	y = 349.87x + 1.36	0.2	0.5	0.67	104.08	9.33	115.91	7.5	115.72	6.01	12.75	16.09	6.09
Sulfadimethoxine	0.2–20	0.9999	y = 1335.77x + 180.94	0.1	0.2	1.08	75.07	18.08	111.01	10.92	100.21	2.73	9.03	5.26	13.62
Sulfadoxine	0.2–20	0.9999	y = 1385.83x + 127.07	0.1	0.2	1.08	111.94	13.19	88.85	9.17	105.11	11.64	16.05	13.89	10.8
Sulfamerazine	0.5–50	0.9993	y = 274.68x + 75.30	0.2	0.5	1.15	91.61	16.38	83.7	12.09	98.59	7.91	10.85	2.04	7.77
Sulfamethazine	0.5–50	0.9998	y = 384.44x + 40.88	0.2	0.5	1.18	81.95	8.36	71.35	5.71	101.25	2.12	14.33	8.15	6.42
Sulfamethizole	0.5–50	0.9998	y = 218.18x + 72.03	0.2	0.5	0.93	71.46	13.24	110.93	5.51	76.55	8.69	15.12	13.32	12.03
Sulfamethoxazole	0.5–50	0.9995	y = 184.66x + 9.45	0.2	0.5	1.07	102.93	8.11	109.87	17.06	92.96	14.74	16.29	5.43	8.52
Sulfamethoxydiazine	0.2–20	0.9998	y = 493.31x + 123.56	0.1	0.2	1.05	77.37	11.51	105.22	5.37	90.82	2.67	14.24	5.78	2.8
Sulfamonomethoxine	0.2–20	0.9997	y = 876.52x + 101.21	0.1	0.2	0.86	90.18	8.46	110.72	4.89	96.33	1.92	10.5	13.59	2.84
Sulfaquinoxaline	0.5–50	0.9996	y = 160.03x + 6.82	0.2	0.5	1.03	116.72	18.77	77.58	16.39	113.79	2.96	5.85	10.68	6.36
Sulfathiazole	0.5–50	0.999	y = 298.00x + 58.03	0.2	0.5	0.84	113.35	17.56	105.29	13.2	93.55	7.92	11.31	8.39	15.7
Sulfisoxazole	0.2–20	0.9992	y = 271.67x − 5.78	0.1	0.2	0.99	96.92	8.18	110.58	6.27	103.93	5.52	19.51	4.33	8.39
Testosterone	1–100	0.9997	y = 145.88x − 24.71	0.3	1	1.49	104.5	14.57	84.66	16.78	115.36	17.69	17.39	12.82	18.01
Tilmicosin	10–1000	0.9973	y = 15.86x − 7.92	3	10	0.94	89.17	3.68	102.51	3.93	79.77	2.18	11.22	13.36	7.15
Toltrazuril	2–200	0.9983	y = 55.46x + 170.66	0.7	2	0.81	93.4	7.04	96.34	3.52	108.5	3.93	15.35	11.29	14.66
Toltrazuril sulfone	2–200	0.9984	y = 85.54x + 211.61	0.7	2	1.67	97.89	12.63	73.44	9.02	72.29	7.5	14.62	7.41	6.09
Toltrazuril sulfoxide	5–500	0.9987	y = 2.81x + 13.72	2	5	2.98	102.58	7.33	116.89	7.06	86.61	6.38	12.9	15.55	15.75
Triamcinolone acetonide	0.5–50	0.9996	y = 259.15x + 36.83	0.2	0.5	1.18	87.72	6.72	120.98	7.49	91.09	4.61	11.18	9.39	5.34
Trimethoprim	0.2–20	0.9999	y = 377.63x + 86.49	0.1	0.2	0.9	91.34	15.5	75.3	2.58	99.45	7.2	10.89	13.03	5.11
Tylosin	0.5–50	0.9996	y = 275.25x − 82.56	0.2	0.5	0.96	97.99	12.19	113.38	10.49	70.81	10.32	13.19	11.9	14.44

^a^ limit of detection. ^b^ limit of quantification. ^c^ matrix effect. ^d^ recovery. ^e^ relative standard deviation.

**Table 2:** Comparison of method performance for contaminants in eggs.

Analysis	Workflow	Cleanup Strategies	Sorbent	Instrument	LOQ (μg/kg)	Recovery (%)	Reference
Fipronil and Fipronil sulfone	Sample→solvent→vortex→saltingout→centrifuge→FaPEx→filter	FaPEx	PSA, C18, MgSO_4_	UPLC-MS/MS	0.1–0.25	88.75–110.91	[2]
25 antiparasitics	Sample→solvent→vortex→saltingout→centrifuge→LTP→centrifuge→concentration	LTP	/	LC-MS/MS	0.22–0.73	87–108	[10]
60 pesticides	Sample→solvent→vortex→saltingout→centrifuge→QuEChERS→vortex→concentration	QuEChERS	Commercialized purification kit	LC-MS/MS and GC-MS/MS	<10	70–120	[17]
74 contaminants	Sample→solvent→vortex→saltingout→centrifuge→QuEChERS→vortex→magnet→concentration	QuEChERS	Fe_3_O_4_-MWCNTs	UPLC-MS/MS	0.1–17.3	60.5–114.6	[24]
31 veterinary drugs	Sample→solvent→vortex→saltingout→centrifuge→dSPE→concentration	dSPE	C18, PSA, NH_2_	UPLC-MS/MS	1–10	61.2–105.7	[40]
13 PBDEs	Sample→solvent→vortex→saltingout→centrifuge→QuEChERS→DLLME→centrifuge	QuEChERS + DLLME	Z-Sep^+^, MgSO_4_	GC-MS/MS	10	85.2–105.7	[41]
59 pesticide	Sample→solvent→vortex→saltingout→centrifuge→dSPE→filter	QuEChERS	PSA, C18, MgSO_4_	LC-MS/MS	5–10	70–120	[42]
60 contaminants	Sample→solvent→vortex→saltingout→centrifuge→SPE→concentration	SPE	PSA, GCB C18, MgSO_4_	LC-MS/MS	0.2–10	70–120	This study

## Data Availability

The original contributions presented in this study are included in the article/Supplementary Materials. Further inquiries can be directed to the corresponding authors.

## Supplementary Materials

The following supporting information can be downloaded at https://www.mdpi.com/article/10.3390/foods15152716/s1, Table S1. The chemical information of 60 analytes determined by LC-MS. Table S2. The MRM parameters of 60 analytes determined by LC-MS. Table S3. The average recovery of all analytes in different extraction solution acidity. Table S4. (a) Three-factor three-level Box-Behnken design. (b) ANOVA results for factors. (c)ANOVA for model.

## Abbreviations

The following abbreviations are used in this manuscript:

ACN	Acetonitrile
AA	Acetic acid
BAGI	Blue Applicability Grade Index
ESI	Electrospray ionization
FA	Formic acid
GCB	Graphitized carbon black
HPLC-MS/MS	High-performance liquid chromatography-tandem mass spectrometry
LOD	Limits of detection
Log P	Log octanol–water partition coefficient
LOQ	Limit of Quantification
LTP	Low-temperature purification
ME	Matrix effect
MeOH	Methanol
MRL	Maximum residue limits
PSA	Primary secondary amine
RSD	Relative standard deviation
RSM	Response surface methodology
SPE	Solid-phase extraction
TICs	Total ion chromatograms
