# Supplementary material for: In-House Fabricated One-Step SPE Cartridge with HPLC-MS/MS for Fast and Accurate Determination of Multi-Class Contaminants in Eggs
Source: Foods. 2026 Aug 1;15(15):2716. doi: 10.3390/foods15152716 (PMC13465489; doi:10.3390/foods15152716)
Supplement: Supplementary file 1 [file foods-15-02716-s001.zip › foods-4463760-supplementary.pdf]

Table S1 The chemical information of 60 analytes determined by LC-MS

Type	Analyte	CAS	Molecular formula	Molecular weight
Amphenicols	Chloramphenicol	56-75-7	$C_{11}H_{12}Cl_2N_2O_5$	323.126
Anthelmintics	Lobendazole	6306-71-4	$C_{10}H_{11}N_3O_2$	205.217
	Levamisole	14769-73-4	$C_{11}H_{12}N_2S$	204.291
Coccidiostats	Diclazuril	101831-37-2	$C_{17}H_9Cl_3N_4O_2$	407.635
	Clopidol	2971-90-6	$C_7H_7Cl_2NO$	192.039
	Toltrazuril	69004-03-1	$C_{18}H_{14}F_3N_3O_4S$	425.381
	Toltrazuril sulfone	69004-04-2	$C_{18}H_{14}F_3N_3O_6S$	457.379
	Toltrazuril sulfoxide	69004-15-5	$C_{18}H_{14}F_3N_3O_5S$	441.38
Fipronil and its metabolites	Fipronil	120068-37-3	$C_{12}H_4Cl_2F_6N_4OS$	437.139
	Fipronil sulfone	120068-36-2	$C_{12}H_4Cl_2F_6N_4O_2S$	453.138
	Fipronil Sulfide	120067-83-6	$C_{12}H_4Cl_2F_6N_4S$	421.14
	Fipronil Desulfinyl	205650-65-3	$C_{12}H_4Cl_2F_6N_4$	389.08
Quinolones	Danofloxacin	112398-08-0	$C_{19}H_{20}FN_3O_3$	357.385
	Enrofloxacin	93106-60-6	$C_{19}H_{22}FN_3O_3$	359.401
	Flumequine	42835-25-6	$C_{14}H_{12}FNO_3$	261.252
	Ciprofloxacin	85721-33-1	$C_{17}H_{18}FN_3O_3$	331.347
	Lomefloxacin	98079-51-7	$C_{17}H_{19}F_2N_3O_3$	351.353
	Norfloxacin	70458-96-7	$C_{16}H_{18}FN_3O_3$	319.336
	Pefloxacin	70458-92-3	$C_{17}H_{20}FN_3O_3$	333.363
	Sarafloxacin	98105-99-8	$C_{20}H_{17}F_2N_3O_3$	385.37
	Ofloxacin	82419-36-1	$C_{18}H_{20}FN_3O_4$	361.373
	Enoxacin	74011-58-8	$C_{15}H_{17}FN_4O_3$	320.324
Glucocorticoids	Betamethasone	378-44-9	$C_{22}H_{29}FO_5$	392.467
	Dexamethasone	50-02-2	$C_{22}H_{29}FO_5$	392.467
	Prednisolone	50-24-8	$C_{21}H_{28}O_5$	360.45
	Hydrocortisone	50-23-7	$C_{21}H_{30}O_5$	362.466
	Triamcinolone acetonide	76-25-5	$C_{24}H_{31}FO_6$	434.504
Macrolides	Azithromycin	83905-01-5	$C_{38}H_{72}N_2O_{12}$	748.996
	Erythromycin	114-07-8	$C_{37}H_{67}NO_{13}$	733.937
	Tylosin	1401-69-0	$C_{46}H_{77}NO_{17}$	916.112
	Tilmicosin	108050-54-0	$C_{46}H_{80}N_2O_{13}$	869.147
	Oleandomycin	3922-90-5	$C_{35}H_{61}NO_{12}$	687.868
Mycotoxins	Aflatoxin B1	1162-65-8	$C_{17}H_{12}O_6$	312.277
	Aflatoxin B2	7220-81-7	$C_{17}H_{14}O_6$	314.293
	Aflatoxin G2	7241-98-7	$C_{17}H_{14}O_7$	330.292
	Aflatoxin G1	1165-39-5	$C_{17}H_{12}O_7$	328.276
Nitroimidazoles	Dimetridazole	551-92-8	$C_5H_7N_3O_2$	141.13
	Metronidazole	443-48-1	$C_6H_9N_3O_3$	171.156
	Ronidazole	7681-76-7	$C_6H_8N_4O_4$	200.154
	Hydroxymetronidazole	4812-40-2	$C_6H_9N_3O_4$	187.155
Quinoxalines	Olaquindox	23696-28-8	$C_{12}H_{13}N_3O_4$	263.253

Type	Analyte	CAS	Molecular formula	Molecular weight
Steroid hormones	Testosterone	58-22-0	$C_{19}H_{28}O_2$	288.431
	Medroxyprogesterone	520-85-4	$C_{22}H_{32}O_3$	344.495
Sulfonamides	Sulfamethazine	57-68-1	$C_{12}H_{14}N_4O_2S$	278.33
	Sulfisoxazole	127-69-5	$C_{11}H_{13}N_3O_3S$	267.303
	Sulfamethizole	144-82-1	$C_9H_{10}N_4O_2S_2$	270.325
	Sulfamethoxazole	723-46-6	$C_{10}H_{11}N_3O_3S$	253.276
	Sulfamerazine	127-79-7	$C_{11}H_{12}N_4O_2S$	264.303
	Sulfadimethoxine	122-11-2	$C_{12}H_{14}N_4O_4S$	310.328
	Sulfamonomethoxine	1220-83-3	$C_{11}H_{12}N_4O_3S$	280.302
	Sulfamethoxydiazine	651-06-9	$C_{11}H_{12}N_4O_3S$	280.302
	Sulfaquinoxaline	59-40-5	$C_{14}H_{12}N_4O_2S$	300.336
	Sulfadoxine	2447-57-6	$C_{12}H_{14}N_4O_4S$	310.328
	Sulfachlorpyridazine	80-32-0	$C_{10}H_9ClN_4O_2S$	284.718
	Sulfadiazine	68-35-9	$C_{10}H_{10}N_4O_2S$	250.276
	Sulfathiazole	72-14-0	$C_9H_9N_3O_2S_2$	255.31
	Trimethoprim	738-70-5	$C_{14}H_{18}N_4O_3$	290.323
β_2-adrenergic agonists	Clenbuterol	37148-27-9	$C_{12}H_{18}Cl_2N_2O$	277.189
	Ractopamine	97825-25-7	$C_{18}H_{23}NO_3$	301.386
	Salbutamol	18559-94-9	$C_{13}H_{21}NO_3$	239.315

Table S2 The MRM parameters of 60 analytes determined by LC-MS

Type	Analyte	RT[a]	log P	Precursor Ion	Product Ion	Ion Mode
Amphenicols	Chloramphenicol	0.916	1.02	321.1	257.1,152.1	Negative
Anthelmintics	Lobendazole	15.399	2.05	334.1	111.0,155.0	Positive
	Levamisole	0.938	1.84	205.1	178.1,123.0	Positive
Coccidiostats	Diclazuril	3.293	2.44	405.0,407.0[b]	334.0,336.0	Negative
	Clopidol	0.987	1.24	192.1	51.0,101.0	Positive
	Toltrazuril	3.193	2.9	424.0	424.0	Negative
	Toltrazuril sulfone	2.446	2.62	456.0	456.0	Negative
	Toltrazuril sulfoxide	1.805	1.81	440.1	383.1,371.1	Negative
Fipronil and its metabolites	Fipronil	3.368	4.75	434.9	330.0,250.0	Negative
	Fipronil sulfone	3.847	7.44	451.0	415.0,282.0	Negative
	Fipronil Sulfide	3.854	6.4	419.0	383.0,314.0	Negative
	Fipronil Desulfinyl	3.568	6.95	387.2	351.1,282.1	Negative
Quinolones	Danofloxacin	7.269	1.2	358.1	340.1,255.0	Positive
	Enrofloxacin	7.534	1.88	360.1	342.0,316.1	Positive
	Flumequine	10.485	2.41	262.1	224.1,202.0	Positive
	Ciprofloxacin	6.86	0.65	332.1	314.1,231.0	Positive
	Lomefloxacin	7.173	1.71	352.1	265.1,308.1	Positive
	Norfloxacin	6.169	0.82	320.0	302.1,276.1	Positive
	Pefloxacin	6.763	1.51	334.1	316.2,290.2	Positive
	Sarafloxacin	8.472	2.09	386.1	368.1,342.1	Positive
	Ofloxacin	1.093	0.84	362.0	261.1,318.1	Positive
	Enoxacin	1.024	0.55	321.0	303.2,232.1	Positive
Glucocorticoids	Betamethasone	13.441	1.87	393.2	373.2,355.2,147.1	Positive
	Dexamethasone	13.441	1.87	393.2	373.2,355.147.1	Positive
	Prednisolone	12.164	1.5	361.2	343.2,147.1	Positive
	Hydrocortisone	12.26	1.43	363.4	121.1,327.2	Positive
	Triamcinolone acetonide	13.898	2.5	435.2	415.2,397.1	Positive
Macrolides	Azithromycin	10.446	3.33	749.5	591.3,158.0	Positive
	Erythromycin	12.882	2.83	734.5	158.0,576.1	Positive
	Tylosin	13.291	3.27	916.5	174.1,772.4,101.0	Positive
	Tilmicosin	12.11	4.95	869.6	174.1,696.4	Positive
	Oleandomycin	12.376	1.23	688.3	158.2,544.3	Positive
Mycotoxins	Aflatoxin B1	12.94	0.45	313.1	285.0,241.0	Positive
	Aflatoxin B2	12.144	0.37	315.0	284.0,259.0	Positive
	Aflatoxin G2	11.275	-0.25	331.0	285.0,245.0	Positive
	Aflatoxin G1	12.192	-0.17	329.0	283.0,243.0	Positive
Nitroimidazoles	Dimetridazole	3.208	0.31	141.2	96.0,81.0	Positive
	Metronidazole	2.797	-0.01	172.1	128.0,820	Positive
	Ronidazole	3.568	-0.45	201.1	140.1,55.2	Positive
	Hydroxymetronidazole	1.567	-0.81	188.0	126.0,123.0	Positive
Quinoxalines	Olaquindox	1.005	-2.2	264.1	143.0, 230	Positive

Type	Analyte	RT[a]	log P	Precursor Ion	Product Ion	Ion Mode
Steroid hormones	Testosterone	14.824	3.48	289.2	109.0,97.0	Positive
	Medroxyprogesterone	16.842	3.38	361.2	163.1,121.1	Positive
Sulfonamides	Sulfamethazine	1.076	0.8	279.1	186.1,156.1	Positive
	Sulfisoxazole	9.905	1.01	268.1	156.0,113.0	Positive
	Sulfamethizole	6.721	0.51	271.0	156.0,108.0	Positive
	Sulfamethoxazole	9.014	0.89	254.1	156.0,108.0	Positive
	Sulfamerazine	4.936	0.34	265.0	156.0,92.0	Positive
	Sulfadimethoxine	8.72	1.48	311.0	156.0,108.0	Positive
	Sulfamonomethoxine	6.551	-0.04	281.1	156.0,108.0	Positive
	Sulfamethoxydiazine	6.816	0.42	281.1	156.0,108.0	Positive
	Sulfaquinoxaline	11.663	1.3	301.1	156.0,108.0	Positive
	Sulfadoxine	8.72	0.34	311.1	156.0,92.0	Positive
	Sulfachlorpyridazine	8.046	1.02	285.0	156.0,108.0	Positive
	Sulfadiazine	3.612	-0.12	251.1	156.0,108.0	Positive
	Sulfathiazole	4.504	0.05	256.0	156.0,108.0	Positive
	Trimethoprim	5.216	0.38	291.1	230.1,123.0	Positive
β_2-adrenergic agonists	Clenbuterol	8.047	2.61	277.0	203.0,168.1	Positive
	Ractopamine	7.104	1.65	302.0	284.0,164.1	Positive
	Salbutamol	1.01	0.01	240.1	148.0,222.1	Positive

[a]RT: retention time

[b]Two precursor ions were monitored for isotopic confirmation due to three chlorine atoms

Table S3 The average recovery of all analytes in different extraction solution acidity

Analytes	ACN	ACN (1%AA)	ACN (2%AA)	ACN (1%FA)	ACN (2%FA)
Aflatoxin B1	75.56	85.61	94.54	77.66	79.87
Aflatoxin B2	67.93	85.83	85.50	75.96	86.48
Aflatoxin G1	56.22	76.71	75.93	74.67	69.69
Aflatoxin G2	53.15	92.07	85.08	77.39	103.26
Azithromycin	85.74	96.39	86.35	74.90	97.19
Betamethasone	84.90	91.17	86.42	74.32	76.50
Chloramphenicol	152.93	89.55	81.71	60.99	51.42
Ciprofloxacin	2.82	42.55	48.59	68.31	70.52
Clenbuterol	76.59	80.93	82.57	81.98	76.61
Clopidol	175.59	151.17	153.70	147.26	152.70
Danofloxacin	6.55	53.81	60.08	60.55	72.98
Dexamethasone	84.90	91.17	86.31	74.32	76.66
Diclazuril	68.25	67.30	72.22	71.70	69.04
Dimetridazole	89.97	103.91	86.73	111.56	105.10
Enoxacin	1.18	25.30	42.60	64.38	73.81
Enrofloxacin	27.44	78.74	78.42	69.08	74.81
Erythromycin	158.37	141.19	179.03	128.85	134.63
Fipronil	91.73	104.34	126.29	116.38	119.44
Fipronil Sulfide	85.39	92.95	107.20	102.04	109.02
Fipronil sulfone	83.69	90.72	98.61	92.09	94.25
Fipronil Desulfinyl	95.98	121.11	145.80	134.57	130.27
Flumequine	33.06	96.34	97.55	93.75	88.64
Hydrocortisone	138.27	98.15	121.60	91.98	131.48
Hydroxymetronidazole	103.77	117.92	75.47	61.32	90.57
Levamisole	80.80	90.42	79.77	86.71	86.71
Lobendazole	73.92	85.05	80.18	76.69	83.67
Lomefloxacin	1.57	49.79	60.42	71.75	76.91
Medroxyprogesterone	100.08	102.58	115.90	96.63	97.42
Metronidazole	107.52	114.28	112.23	112.55	107.08
Norfloxacin	1.48	25.50	30.88	68.41	74.72
Ofloxacin	10.50	62.08	60.60	67.81	74.89
Olaquindox	66.67	123.10	126.07	91.75	79.21
Oleandomycin	92.55	103.13	101.12	99.58	92.41
Pefloxacin	14.01	63.87	62.34	60.37	73.23
Prednisolone	77.89	88.78	91.62	86.10	68.68
Ractopamine	74.23	80.87	80.71	79.84	76.94
Ronidazole	72.25	90.86	101.94	86.69	90.11
Salbutamol	39.12	82.23	46.70	67.66	68.94
Sarafloxacin	6.41	55.47	70.36	69.93	76.82
Sulfachlorpyridazine	62.36	96.74	81.56	86.57	102.89
Sulfadiazine	65.50	97.80	96.98	96.41	111.09
Sulfadimethoxine	87.42	94.88	106.78	99.73	92.91

Sulfadoxine	87.42	94.88	106.78	99.73	92.91
Sulfamerazine	68.67	85.04	88.94	79.38	79.56
Sulfamethazine	84.77	90.00	82.84	92.71	87.10
Sulfamethizole	62.79	100.29	95.64	97.09	105.96
Sulfamethoxazole	80.75	97.87	122.09	99.49	95.04
Sulfamethoxydiazine	97.48	102.52	91.71	101.23	89.69
Sulfamonomethoxine	76.06	86.11	86.84	84.81	88.81
Sulfaquinoxaline	69.76	86.74	88.29	102.96	91.38
Sulfathiazole	87.13	94.06	94.17	101.65	97.47
Sulfisoxazole	83.55	91.45	105.37	115.24	98.36
Testosterone	85.07	90.80	79.53	88.45	87.98
Tilmicosin	84.11	123.18	121.19	99.34	80.13
Toltrazuril	53.64	33.69	30.93	59.99	56.62
Toltrazuril sulfone	47.13	33.09	27.06	37.39	59.87
Toltrazuril sulfoxide	60.92	42.02	34.62	42.04	67.07
Triamcinolone acetonide	70.45	88.95	76.21	75.68	73.59
Trimethoprim	76.11	83.04	72.30	67.47	76.11
Tylosin	83.65	112.35	106.75	107.06	93.51

Table S4

(a) Three-factor three-level Box-Behnken design

Level	A/PSA (mg)	B/GCB (mg)	C/C18 (mg)
-1	100	20	200
0	200	40	300
1	300	60	400

(b)ANOVA results for factors

Reponse	Factors	Sum of squares	Degree of freedom	Mean squares	F-value	P-value
Y	A	26.04	1	26.04	8.66	0.0216
	B	63.37	1	63.37	21.07	0.0025
	C	84.84	1	84.84	28.21	0.0011
	AB	4.00	1	4.00	1.33	0.2866
	AC	2.25	1	2.25	0.7482	0.4157
	BC	6.25	1	6.25	2.08	0.1926
	A^2	279.67	1	279.67	93.00	<0.0001
	B^2	159.25	1	159.25	52.96	0.0002
	C^2	81.52	1	81.52	27.11	0.0012

(c)ANOVA for model

Reponse	Degree of freedom	R^2	F-value	P-value	Adeq Precision	Coefficient value (%)	Lack of fit p-value
Y	9	0.9781	34.69	<0.0001	16.3909	3.47	0.1731
